# The recommended dosage regimen for caspofungin in patients with higher body weight or hypoalbuminaemia will result in low exposure: Five years of data based on a population pharmacokinetic model and Monte-Carlo simulations

**DOI:** 10.3389/fphar.2022.993330

**Published:** 2022-11-03

**Authors:** Qianting Yang, Tao Zhang, Ying Zhang, Dan Sun, Xiaowei Zheng, Qian Du, Xue Wang, Xiaoliang Cheng, Jianfeng Xing, Yalin Dong

**Affiliations:** ^1^ Department of Pharmacy, The Second Affiliated Hospital of Xi’an Jiaotong University, Xi’an, China; ^2^ Department of Pharmacy, The First Affiliated Hospital of Xi’an Jiaotong University, Xi’an, China; ^3^ Department of Pharmacy, Xi’an Hospital of Traditional Chinese Medicine, Xi’an, China; ^4^ Department of Pharmacy, Xi’an No.1 Hospital, Xi’an, China; ^5^ Department of Intensive Care Unit, The First Affiliated Hospital of Xi’an Jiaotong University, Xi’an, China; ^6^ School of Pharmacy, Xi’an Jiaotong University, Xi’an, China

**Keywords:** caspofungin, population pharmacokinetics, intensive care unit, body weight, hypoalbuminaemia

## Abstract

**Background:** To develop a population pharmacokinetic (PPK) model for caspofungin, identify parameters influencing caspofungin pharmacokinetics, and assess the required probability of target attainment (PTA) and cumulative fraction of response (CFR) for various dosing regimens of caspofungin in all patients and intensive care unit (ICU)-subgroup patients.

**Method:** The general PPK model was developed based on data sets from all patients (299 patients). A ICU-subgroup PPK model based on data sets from 136 patients was then analyzed. The effects of demographics, clinical data, laboratory data, and concomitant medications were tested. Monte-Carlo simulations (MCS) were used to evaluate the effectiveness of different caspofungin dosage regimens.

**Results:** One-compartment model best described the data of all patients and ICU patients. Clearances (CL) were 0.32 L/h and 0.40 L/h and volumes of distribution (V) were 13.31 L and 10.20 L for the general and ICU-subgroup PPK models, respectively. In the general model, CL and V were significantly associated with albumin (ALB) concentration and body weight (WT). In the ICU-subgroup model, CL was associated with WT. The simulated exposure in ICU patients was lower than that in all patients (*p* < 0.05). MCS indicated that higher caspofungin maintenance doses of 70–150 mg may achieve target CFR of >90% for patients with higher WT (>70 kg) or with *C. albicans* or *C. parapsilosis* infections, and especially for ICU patients with hypoalbuminaemia.

**Conclusion:** The PPK model and MCS presented in the study demonstrated that the recommended dosage regimen for caspofungin in patients with higher body weight or hypoalbuminaemia will result in low exposure.

## 1 Introduction

Caspofungin is the first echinocandin approved for the treatment of invasive fungal infections (IFIs) caused by *Candida* and *Aspergillus* spp. In patients who are refractory to or intolerant of azole antifungal agents (Pappas et al., 2009). The recommended dosage regimen for caspofungin is a loading dose of 70 mg followed by 50 mg daily (70/50 mg) (Pappas et al., 2009). However, a reduced or increased dosage regimen is recommended for patients with liver dysfunction or a higher body weight (WT) (Muilwijk et al., 2015). Intensive care unit (ICU) patients are susceptible to fungal infections. The good tolerance to caspofungin and its weak interactions with other drugs (Denning, 2003) make caspofungin a potentially important agent in the treatment of IFIs in ICU patients (Nguyen et al., 2007). IFIs following solid-organ transplant (SOT) is associated with significant morbidity and mortality (Petrovic et al., 2007); this is also the case for hematopathy (HEMT) patients (Wurthwein et al., 2013). Antifungal treatment and prophylaxis are rational for these patients with a high IFI risk, and caspofungin appears to be an effective and well-tolerated option for them (Wurthwein et al., 2013).

Large inter- and intraindividual variabilities have been observed in the plasma concentrations of caspofungin for the recommended dosage regimen, with the plasma trough concentration (*C*_min_) ranging from 0.52 to 4.08 mg/L in ICU patients (Nguyen et al., 2007), thereby varying markedly compared to those observed in healthy subjects (1.12–1.78 mg/L) (Stone et al., 2002). Van den Elst et al. found the caspofungin exposure in ICU patients was low compared with that in healthy volunteers and other (non)critically ill patients (van der Elst et al., 2017). Many factors could influence the caspofungin plasma concentrations. WT and sex influence caspofungin plasma concentrations in healthy subjects (Nguyen et al., 2007). Trauma, surgery and sepsis could induce physiological and physiopathological alterations in ICU patients and could lead to changes in the pharmacokinetics (PK) of caspofungin (Nguyen et al., 2007). Increasing clearance with subsequent doses was associated with a clinically relevant decrease in caspofungin exposure (>20%) in ICU patients (Borsuk-De Moor et al., 2020). Li et al. found that blood albumin (ALB) and total bilirubin levels were factors affecting caspofungin clearance (CL), while WT was the only factor affecting volume of distribution (V) among Chinese people with relatively low weight compared with other populations (Li et al., 2021). To date, there is limited literature on the PK variability of caspofungin in general patients. Although some studies have considered the variability of caspofungin plasma concentrations in ICU patients, they did not compare the PK variability of caspofungin in ICU patients and the general patients. The large inter- and intra-individual variability in caspofungin plasma concentrations among different patients makes it necessary to study the population pharmacokinetics (PPK) of caspofungin in all patients, especially in the ICU patients.

The activity of caspofungin against *Candida* spp. Is concentration-dependent and correlated with the area under the plasma concentration-time curve divided by the minimum inhibitory concentration (AUC/MIC) (Louie et al., 2005; Andes et al., 2010). In order to estimate the feasibility and effectiveness of the recent dosage regimens for caspofungin in all patients, and especially ICU patients, it is necessary to evaluate the caspofungin PK variability as well as determine the MIC. Previous study based on murine models by Andes et al. provided the standard recommendations to predict the PD targets of *C. albicans*, *C. glabrata* and *C. parapsilosis* (Andes et al., 2010). Then a Monte-Carlo simulations (MCS) method could be used as a tool to link the above information to determine dosage regimens and select the more appropriate empirical caspofungin treatments at the national, regional and institutional levels.

The current study performed a PPK analysis with MCS in patients with Candida infections or suspected fungal infections receiving caspofungin with the aim of describing the PK characteristics of this drug, determine factors influencing caspofungin PK variability, identify the effectiveness of the recent caspofungin dosage regimens, and establish more-reasonable individualized dosage regimens for caspofungin in all patients and ICU patients.

## 2 Materials and methods

### 2.1 Patients and inclusion criteria

The patients for the general PPK model came from a single-center, and the study was conducted from June 2014 to June 2019 at the First Affiliated Hospital of Xi’an Jiaotong University. Patients who had been diagnosed with confirmed or probable candidiasis and been treated with caspofungin were included in the study. The identification of infection was according to the IFIs Cooperative Group and the National Institute of Allergy and Infectious Diseases Mycoses Study Group criteria (De Pauw et al., 2008). All patients were divided into four groups (ICU patients, SOT patients, HEMT patients, and other patients from respiratory department). Caspofungin was administered intravenously over 1 h. Most of the patients received the standard dosage regimen of 70/50 mg. Patients with hepatic insufficiency (Child-Pugh B) received a reduced dosage regimen of 70/35 mg and patients with body weight (WT) > 75 kg received an increased dosage regimen of 70/70 mg. The study protocol was approved by the institutional review board of the teaching hospital (No.XJTU1AF2017LSK-28). All subjects signed the informed consent before any screening item being performed. History of hypersensitivity, severe intolerance to caspofungin, other constituents change resulted from caspofungin, age <18 years and pregnancies were excluded in the study.

The subgroup PPK model of ICU patients (ICU-subgroup PPK model) included 51 ICU patients from the First Affiliated Hospital of Xi’an Jiaotong University, Shaanxi, China, and 85 ICU patients from other seven studies (Spriet et al., 2009; Spriet et al., 2011; Weiler et al., 2013; Muilwijk et al., 2014; Sinnollareddy et al., 2015; Roger et al., 2017; van der Elst et al., 2017). The dosage regimen given to ICU patients was the same as that given to the general patients.

### 2.2 Clinical data collection

Factors which may influence the PK parameters of caspofungin were collected for the patients of the general PPK model from the teaching hospital. The factors included demographic factors (sex, age and WT); medical characteristics [carrier mediated transportation (CMT), surgical operation (SOP), baseline disease, organ support therapy]; laboratory data [red blood cells, hemoglobin, hematocrit, white blood cell, lymphocyte, neutrophile, platelets, alanine aminotransferase, aspartate transaminase, alkaline phosphatase, gamma-glutamyl transpeptidase, total bilirubin, albumin (ALB), blood urea nitrogen, uric acid, serum creatinine acid, creatinine clearance rate]; concomitant medications (immunosuppressants, glucocorticoid, antimicrobial agents and antivirals) and other related factors. Because we could not obtain all values of ICU patients for the seven literature studies, only factors which may have a great influence on the PK of caspofungin, such as sex, age, WT, dosage and method of administration, ALB concentrations and whether concomitant continuous renal replacement therapy (CRRT) of ICU patients from the teaching hospital and other seven studies were collected for the construction of the ICU-subgroup PPK model. The selection criteria were based on the results obtained from previous studies of factors influencing the caspofungin plasma concentration (Spriet et al., 2009; Spriet et al., 2011; Weiler et al., 2013; Muilwijk et al., 2014; Sinnollareddy et al., 2015; Roger et al., 2017; van der Elst et al., 2017). In case the author did not provide the exact values, few data were replaced by missing values. Combined with the clinical situation, the ALB and WT were converted into dichotomous variables according to whether the ALB <35 g/L or ≥35 g/L and whether the WT < 70 kg or ≥70 kg, respectively.

### 2.3 Blood sample collection and analysis

We collected the pooled data of caspofungin plasma samples from patients at the teaching hospital who received caspofungin prevention or treatment therapies from June 2014 to June 2019. Caspofungin plasma *C*_min_ samples were collected at interval windows of 22–24 h post-dose, while other caspofungin plasma samples were collected at interval windows of 0–12 h and 12–22 h post-dose. The caspofungin plasma concentrations from the other seven studies of ICU patients—which included both dense sampling and sparse sampling—were directly collected from the relevant published papers, and all related caspofungin plasma concentrations were collected (Spriet et al., 2009; Spriet et al., 2011; Weiler et al., 2013; Muilwijk et al., 2014; Sinnollareddy et al., 2015; Roger et al., 2017; van der Elst et al., 2017).

### 2.4 Population pharmacokinetic model

A non-linear mixed-effects population approach with the NONMEM software (version 7.20, Icon Development Solutions, Ellicott City, United States) was used in the full study. We built the caspofungin PPK structural model *via* comparing one- or two-compartment model with zero- and first-order elimination and Michaelis-Menten elimination method. The typical population values of caspofungin CL and V were estimated. Then we considered caspofungin PPK statistical model, the exponential error model was used to evaluate the interindividual variability of the PK parameters.

Exponential error model: ${Cobs=C}_{pred}{\times \exp}^{\epsilon }$ C_obs_ and C_pred_ represent the observed and predicted concentrations, respectively. *e* is normal random variable with mean of 0 and variance of *σ*.

The study established the covariate model to identify factors influencing caspofungin PK. The factors which could potentially influence caspofungin pharmacokinetics were considered into the model, and factors that most likely to influence the PK of caspofungin were initially evaluated, such as WT, ALB, concomitant medication of immunosuppressants. They were selected mainly based on the literature (Nguyen et al., 2007; Yang et al., 2020; Li et al., 2021) or the metabolic characteristics of caspofungin in the body. The base model was used to test all of those covariates. If the objective function value (OFV) has a reduction of >3.84 (*p* < 0.05), the covariate added was considered in the base model. If the OFV value decreases greater than 6.84 (*p* < 0.01), the covariates were added one by one to obtain the full-volume model. Based on the full model, a more rigorous statistical standard (ΔOFV >10.83, *p* < 0.001) was used to reverse the covariates to obtain the final model. The specified values of ΔOFV for model selection are for one degree of freedom assuming a chi-squared distribution. PsN (Version 3.4.2, Uppsala University, Sweden) was applied for model construction. The final model was obtained after removing the covariates without significant influence. The optimal model conformed to the following standards: (i) the residual error was small compared to the base model and the OFV was minimized; (ii) the goodness of fit (GOF) was improved; and (iii) the reserved covariates had clinical significance. Both the general PPK model and ICU-subgroup PPK model were established based on the above method.

### 2.5 Model evaluation

For the two PPK models, the robustness and precision of the final model was evaluated using a non-parametric bootstrap method in which the dataset was repeated 1,000 times to produce a new dataset of the same size in the process. A prediction-corrected visual predictive check (pcVPC) was carried out to verify the centralized tendency and variability in the observed data. PsN (Version 3.4.2, Uppsala University, Sweden) was used for model validation and the diagnostic plots were conducted using R (Version 12.2.2).

### 2.6 Different caspofungin dosing simulations

The final general and ICU-subgroup PPK models of caspofungin were used for the simulation study to evaluate the degree of exposure. A MCS method published before (Pfaller et al., 2006) was used for analyzing the probability of target attainment (PTA) and cumulative fraction of response (CFR) following various caspofungin dosage regimens in all patients and ICU patients. The MCS method was used to combine the variability of PPK parameters and MIC data (Pfaller et al., 2013) to determine the PTA and CFR of *f*AUC_24_/MIC ≥20, *f*AUC_24_/MIC ≥7 and *f*AUC_24_/MIC ≥7 for *C. albicans, C. parapsilosis* and *C. glabrata* (Andes et al., 2010), respectively. These AUC/MIC ratios were set as preclinical PK targets to attain the data for the current study. To calculate the free drug concentrations, protein binding value of 97% for caspofungin was used in the simulation (Andes et al., 2010). MCS was used to analyze the mean values and interindividual variances of the population parameters (CL). The CL of caspofungin were obtained from the final general and ICU-subgroup PPK models, then the AUC_24_ were obtained based on the following formula (Fernandez de Gatta Mdel et al., 2009):

$${AUC}_{24}=Dose/CL$$

Based on the simulation above, we could get the AUC_24_ for intravenously administered of caspofungin in all patients and ICU patients.

Five different caspofungin dosage regimens included (I) the recommended dosage regimens of 70/50 mg (II) 70/35 mg in patients with moderate or severe hepatic dysfunction (Pappas et al., 2009); and alternative regimens included (III) 70/70 mg (IV) 100/100 mg (V)150/150 mg were simulated for all patients and ICU patients with WT < 70 kg or ≥70 kg in this study. The PTA at steady state following different dosage regimens was assessed for a wide range of clinically relevant minimum inhibitory concentration (MIC) values (0.008–8 mg/L). The MIC data for *Candida* spp. were obtained from Pfaller et al. (Pfaller et al., 2013). The MCS was performed with 10,000 replicates. The result of MCS was expressed as the PTA and CFR (Mouton et al., 2005). The simulated PTA and CFR of these subjects were compared to choose the optimal dosage regimens. PTA and CFR values equal to and greater than 90% was considered valid in the corresponding MIC condition, as shown in the OPTAMA programme established earlier (Masterton et al., 2005).

## 3 Results

### 3.1 Patient characteristics, samples and dosing

#### 3.1.1 Data for all patients

From June 2014 to June 2019, 921 caspofungin plasma samples (median of 3) from 299 hospitalized patients were measured by a rapid and sensitive liquid chromatography–tandem mass spectrometry method described previously (Yang et al., 2015). The mean recovery rate ranged from 85.2% to 95.3%, while the intra- and inter-day precisions were <5.5%, and their accuracies were within the range of 96.2%–102.3% (Yang et al., 2015). There were 242 caspofungin plasma *C*_min_ samples, and 247 and 432 caspofungin plasma samples collected at interval windows of 0–12 h and 12–22 h post-dose, respectively. Hypoalbuminaemia (<35 g/L) was present in 161 of the 299 hospitalized patients. The demographic and clinical data of the estimated covariates are shown in Table 1. The detailed information are shown in Supplementary Appendix 3.1.1.

**TABLE 1:** Demographics and clinical data for all patients^a^ from the teaching hospital and intensive care unit-subgroup patients^b^ to develop the caspofungin population pharmacokinetic model.

Parameter^c^	Value, mean ± SD (range)			
	All patients from the teaching hospital (*n* = 299)	ICU-subgroup patients (*n* = 136)	ICU patients from the teaching hospital (*n* = 51)	ICU patients from the seven literatures (n = 85)
Age (years)	44 ± 17 (18–99)	60.5 ± 16.2 (23.0–99.0)	65.2 ± 19.4 (23.0–99.0)	54.3 ± 13.5 (25.0–83.0)
WT (kg)	62.3 ± 11.5 (30.0–100.0)	68.1 ± 12.7 (30.0–139.0)	60.7 ± 11.5 (30.0–86.0)	78.7 ± 6.1 (48.0–139.0)
HGB (g/L)	93.2 ± 39.6 (42.3–1106.0)	—	—	—
ALB (g/L)	35.6 ± 11.6 (15.1–64.5)	28.0 ± 6.9 (15.1–64.5)	30.0 ± 8.3 (15.1–64.5)	26.8 ± 5.1 (20.0–35.5)
TBIL (mol/L)	23.6 ± 57.1 (0.4–598.7)	—	—	—
AST (U/L)	34.9 ± 125.5 (1.6–2476.4)	—	—	—
ALP (U/L)	96.8 ± 103.9 (22–1236)	—	—	—
ALT (U/L)	39.6 ± 219.9 (0.7–7144)	—	—	—
PLT (10^9^/L)	124.4 ± 86.8 (0.1–498.0)	—	—	—
CREA (mmol/L)	250.6 ± 248.8 (6.0–1320.0)	—	—	—
CL_CR_ (ml/min)	64.8 ± 84.3 (2.0–1125.0)	—	—	—
**Covariate (no. of patients)**				
Sex (male/female)	207/92	83/53	33/18	50/35
ICU: SOT: HEM: others	51:163:42:43	—	—	—
CMT (intravenous drip/infusion pump)	253/46	—	—	—
CRRT (yes/no)	50/249	50/86	14/37	36/49
SOP (yes/no)	139/160	—	—	—
**Concomitant medication, no. (%) of patient**				
CYC	24 (8.0%)	—	—	—
TAC	29 (9.7%)	—	—	—
MM	91 (30.4%)	—	—	—
MET	110 (36.8%)	—	—	—

ALB, albumin; AST, aspartate transaminase; ALP, alkaline phosphatase; ALT, alanine aminotransferase; CREA, serum creatinine acid; CL_CR_, creatinine clearance rate; CMT, carrier mediated transport; CYC, cyclosporine; HGB, haemoglobin; ICU, intensive care unit; SOT, solid organ transplant; HEM, hematology; MM, mycophenolate mofetil; MET, methylprednisolone; PLT, platelets; CRRT, continuous renal replacement therapy; SOP, surgical operation; TBIL, total bilirubin; TAC, tacrolimus; WT, weight.

^a^ All patients include ICU, patients, transplant patients, hematopathy patients and other patients from the teaching hospital.

^b^ The intensive care unit-subgroup patients include patients from the teaching hospital and the seven literatures.

^c^ Partial of the covariates are listed.

#### 3.1.2 Data for ICU patients

The demographic and clinical data of the estimated covariates for all ICU patients are shown in Table 1. The samples and dosing information are shown in Table 1 and Supplementary Appendix 3.1.2.

### 3.2 PPK model

#### 3.2.1 General PPK model

The data we collected could be fully described by a one-compartment model of first-order absorption and elimination. We tested each covariate in univariate fashion using the base model. Covariates whose addition resulted in a significant reduction in OFV (ΔOFV) of >3.84 were reserved. Table 2 and Supplementary Appendix 3.2.1 show the individual covariates for CL and V according to the base model. The forward inclusion steps were used to select the full model. Supplementary Appendix 3.2.1 shows the selected covariates in the full model. The backward elimination method resulted in the final model containing ALB and WT as significant covariates for both CL and V. The OFV decreased by 135.35 when comparing the final model with the base model.

**TABLE 2:** Individual significant covariates screened with NONMEM for all patients^a^ from the teaching hospital and intensive care unit-subgroup patients^b^.

Parameter	Significant covariate	All patients from the teaching hospital (n = 299)				ICU-subgroup patients^c^ (n = 136)			
		Forward inclusion step		Backward elimination step		Forward inclusion step		Backward elimination step	
		ΔOFV	*p* Value	ΔOFV	*p* Value	ΔOFV	*p* Value	ΔOFV	*p* Value
CL	ALB	−77.87	<0.00001	−77.87	<0.00001	−4.8	<0.05	−4.8	<0.05
	MM	−23.48	<0.00001	−5.27	<0.05	—	—	—	—
	CYC	−20.41	<0.001	−9.20	<0.01	—	—	—	—
	MET	−19.35	<0.001	−5.89	<0.05	—	—	—	—
	WT	−14.91	<0.001	−13.11	<0.001	−11.02	<0.001	−11.02	<0.001
	CMT	−14.31	<0.001	−7.28	<0.01	—	—	—	—
	SOP	−12.92	<0.001	−4.23	<0.05	—	—	—	—
	SOT	−11.19	<0.001	−3.16	<0.1	—	—	—	—
	ICU	−8.49	<0.05	−5.27	<0.05	—	—	—	—
V	ALB	−33.40	<0.00001	−33.40	<0.00001	—	—	—	—
	WT	−20.99	<0.00001	−17.61	<0.001	—	—	—	—
	CMT	−6.96	<0.05	−5.10	<0.05	—	—	—	—

ALB, albumin; MM, mycophenolate mofetil; CYC, cyclosporine; MET, methylprednisolone; WT, weight; CMT, carrier mediated transport; SOP, surgical operation; SOT, solid organ transplantation; ICU, intensive care unit.

^a^ All patients include ICU, patients, transplant patients, hematopathy patients and other patients from the teaching hospital.

^b^ The intensive care unit patients include patients from the teaching hospital and the seven literatures.

^c^ The ΔOFV, and *p* value are the same of forward inclusion step and backward elimination step for ICU, patients.

**FIGURE 1:**
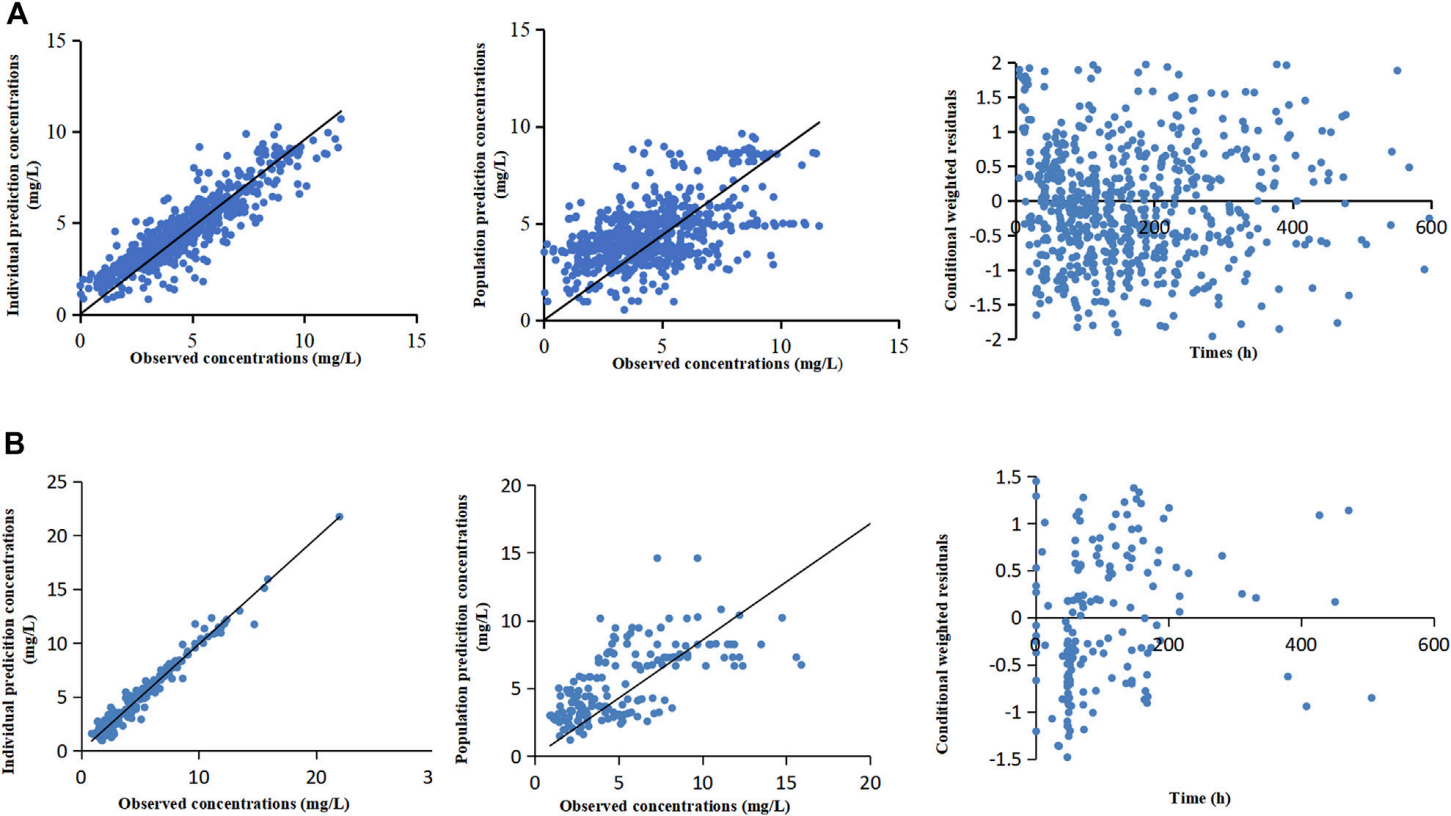
The final population pharmacokinetic models of caspofungin based on all patients from the teaching hospital **(A)** and intensive care unit-subgroup patients **(B)**. The diagnostic scatterplots of the caspofungin population pharmacokinetic models from left to right are as follows: individual prediction concentrations *versus* observed caspofungin plasma concentrations; population prediction concentrations *versus* observed caspofungin plasma concentrations; and conditional weighted residuals *versus* time. The diagonal lines in the upper panels represent lines of unity.

Table 3 lists the relevant estimates of CL, V, and interindividual and residual variabilities of the final model and the base model. Figure 1A shows the final PPK model of caspofungin based on the 299 patients. Figure 1A shows that the individual prediction concentrations provided a good fit to the observed plasma caspofungin concentrations. The population prediction concentrations were improved significantly in the final model compared the base model (Supplementary Figure S1). The individual weighted residuals were uniformly distributed between –1.5 and 1.5 in the final model, which indicated that the error model was suitable in the final general PPK model.

**TABLE 3:** Comparison of the caspofungin pharmacokinetic parameters estimated of the final model and bootstrap analysis for all patients^a^ from the teaching hospital and intensive care unit-subgroup patients^b^.

Patients group	—	Parameter						Inter-individual variability (%)		Residual variability
	—	θ_1_	θ_2_	θ_3_	θ_4_	θ_5_	θ_6_	CL (L/h)	V (L)	Proportional (%)
All patients from the teaching hospital (n = 299)	Final model^c^	0.32	13.31	0.46	0.49	0.98	0.24	29.2	59.2	19.3
	Mean values of bootstrap results	0.31	13.13	0.45	0.48	1.02	0.23	29.0	56.1	18.7
	Lower boundary of 95% CI	0.29	10.18	0.33	0.25	0.30	0.10	20.5	40.3	16.5
	Upper boundary of 95% CI	0.34	16.42	0.59	0.73	1.65	0.37	36.7	67.8	20.4
ICU-subgroup patients (n = 136)	Final model^d^	0.42	10.20	0.34	—	—	—	13.5	19.6	24.3
	Mean values of bootstrap results	0.40	10.50	0.33	—	—	—	12.4	21.2	26.8
	Lower boundary of 95% CI	0.32	7.70	0.11	—	—	—	5.3	4.3	28.6
	Upper boundary of 95% CI	0.46	19.00	0.70	—	—	—	17.7	51.6	34.1

^a^ All patients include ICU, patients, transplant patients, hematopathy patients and other patients from the teaching hospital.

^b^ The intensive care unit patients include patients from the teaching hospital and the seven literatures.

^c^ Final model for all patients from the teaching hospital: θ = population mean parameters and are numbered according to the following equations in the final model: CL = θ_1_ × (1 + θ_3_ × ALB*) × (1 + θ_5_ × WT*) × e^η1^; V = θ_2_ × (1 + θ_4_ × ALB*) × (1 + θ_6_× WT*) × e^η2^. ALB* and WT* are not continuous variables but categorical variables in the two formulas. We defined ALB* = 1 if the patient’s albumin concentration <35 g/L, if not, ALB* = 0; we defined that WT* = 1 if the patient’s weight ≥70 kg, if not, WT* = 0. According to the model, caspofungin plasma concentrations were predicted to be lower in patients with albumin concentration <35 g/L and patients with body weight ≥70 kg θ_1_ and θ_2_ are the typical population values of CL, and V. θ_3_ and θ_4_ are the typical population values of ALB, for the influence on CL, and V, respectively. θ_5_ and θ_6_ are the typical population values of WT, for the influence on CL, and V, respectively.

^d^ Final model for intensive care unit patients: θ = population mean parameters and are numbered according to the following equations in the final model: CL = θ_1_ × (1 + θ_3_ × WT*) × e^η1^; V = θ_2_ × e^η2^. θ_1_ and θ_2_ are the typical population values of CL, and V. θ_3_ is the typical population value of WT, for the influence on CL.

#### 3.2.2 ICU-subgroup PPK model

The ICU-subgroup model could also be well described by a one-compartment model. It was found that WT and ALB had significant impacts on CL (Table 2). However, only WT was saved in the final model after a rigorous statistical standard (ΔOFV >10.83, *p* < 0.001, Table 3), and the OFV decreased by 11.02 when comparing the final model with the base model. Figure 1B shows that the individual prediction concentrations and population prediction concentrations provided good fits to the observed plasma caspofungin concentrations (Supplementary Figure S1). The individual weighted residuals were uniformly distributed between –1.5 and 1.5 in the final ICU-subgroup PPK model (Figure 1B).

### 3.3 Model evaluation

**FIGURE 2:**
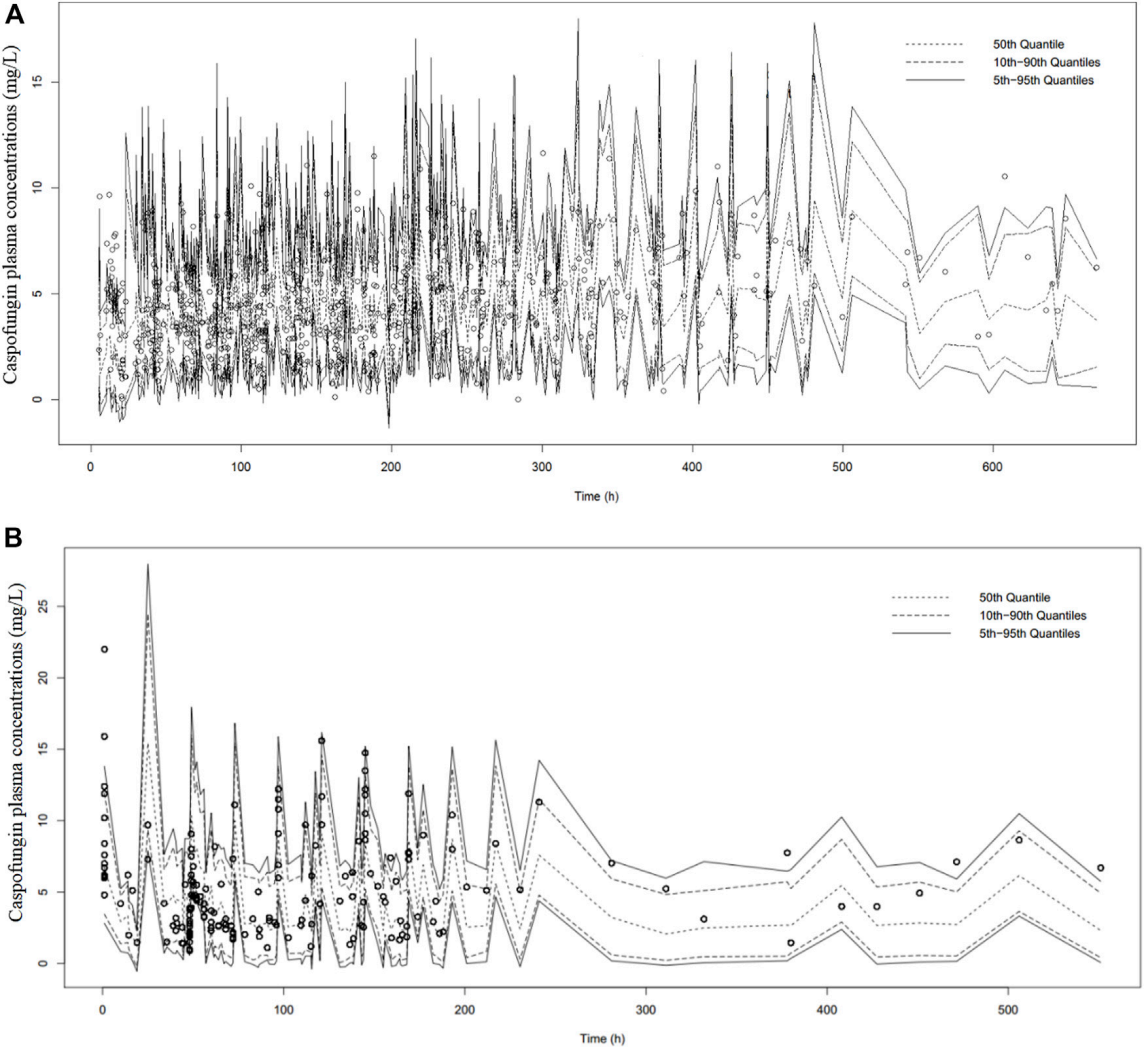
Prediction-corrected visual predictive check of caspofungin in all patients from the teaching hospital **(A)** and intensive care unit-subgroup patients **(B)** based on n = 1000 simulations. The median (short dotted line), 10th and 90th percentiles (long dotted lines) and 5% and 95% percentiles (solid black lines) based on simulations are shown. Individual points represent observed data.

The bootstrap analysis showed that 867 and 895 runs out of 1000 performed and converged successfully for the general and ICU-subgroup PPK final models, respectively. Detailed information are shown in Supplementary Appendix 3.3. The results of the pcVPC are shown in Figure 2. The pcVPC figures provide evidence that the developed PPK models are appropriate to describe the time course of plasma caspofungin concentrations in the present patient groups.

### 3.4 Pharmacokinetics of caspofungin in all patients and ICU patients

#### 3.4.1 Pharmacokinetics of caspofungin in all patients

Table 4 shows the AUC, CL and relative risk factors for caspofungin in four patient groups (ICU patients, SOT patients, HEMT patients and other patients) based on the general PPK model. The mean AUC in ICU patients, SOT patients, HEMT patients and other patients were 101.46, 135.74, 117.75 and 119.60 mgh/L, respectively. Detailed information is shown in Supplementary Appendix 3.4.1.

**TABLE 4:** Pharmacokinetics and clinical data of caspofungin for all patients^a^ from the teaching hospital and intensive care unit-subgroup patients^b^.

Patients group	Mean ± SD (range)	AUC (mg·h/L)	CL (L/h)	WT (kg)	ALB (g/L)
All patients from the teaching hospital (n = 299)	All patients	126.46 ± 45.66 (36.73–303.62)	0.42 ± 0.12 (0.16–0.71)	62.26 ± 11.51 (30–100)	35.57 ± 11.62 (15.07–64.5)
	ICU patients	101.46 ± 43.99 (40.37–265.83)	0.47 ± 0.12 (0.19–0.65)	60.87 ± 12.01 (30–87)	30.02 ± 7.81 (15.07–64.5)
	Transplant patients	135.74 ± 43.96 (43.54–302.88)	0.40 ± 0.12 (0.17–0.71)	62.10 ± 11.41 (38–100)	38.77 ± 10.72 (24.7–59.9)
	Hematopathy patients	117.75 ± 34.40 (41.18–239.99)	0.45 ± 0.11 (0.21–0.65)	66.60 ± 11.91 (46–96)	29.56 ± 12.44 (18.9–55)
	Other patients	119.60 ± 51.35 (36.73–303.62)	0.41 ± 0.11 (0.16–0.68)	60.49 ± 9.97 (44–95)	31.64 ± 11.34 (19.55–42)
ICU-subgroup patients (n = 136)	ICU patients from the teaching hospital and the seven literatures	101.83 ± 33.45 (55.69–193.30)	0.56 ± 0.22 (0.25–1.26)	68.07 ± 12.66 (30–91)	28.02 ± 6.89 (15.07–64.5)

^a^ All patients include ICU, patients, transplant patients, hematopathy patients and other patients from the teaching hospital.

^b^ The intensive care unit patients include patients from the teaching hospital and the seven literatures.

#### 3.4.2 Pharmacokinetics of caspofungin in ICU patients

**FIGURE 3:**
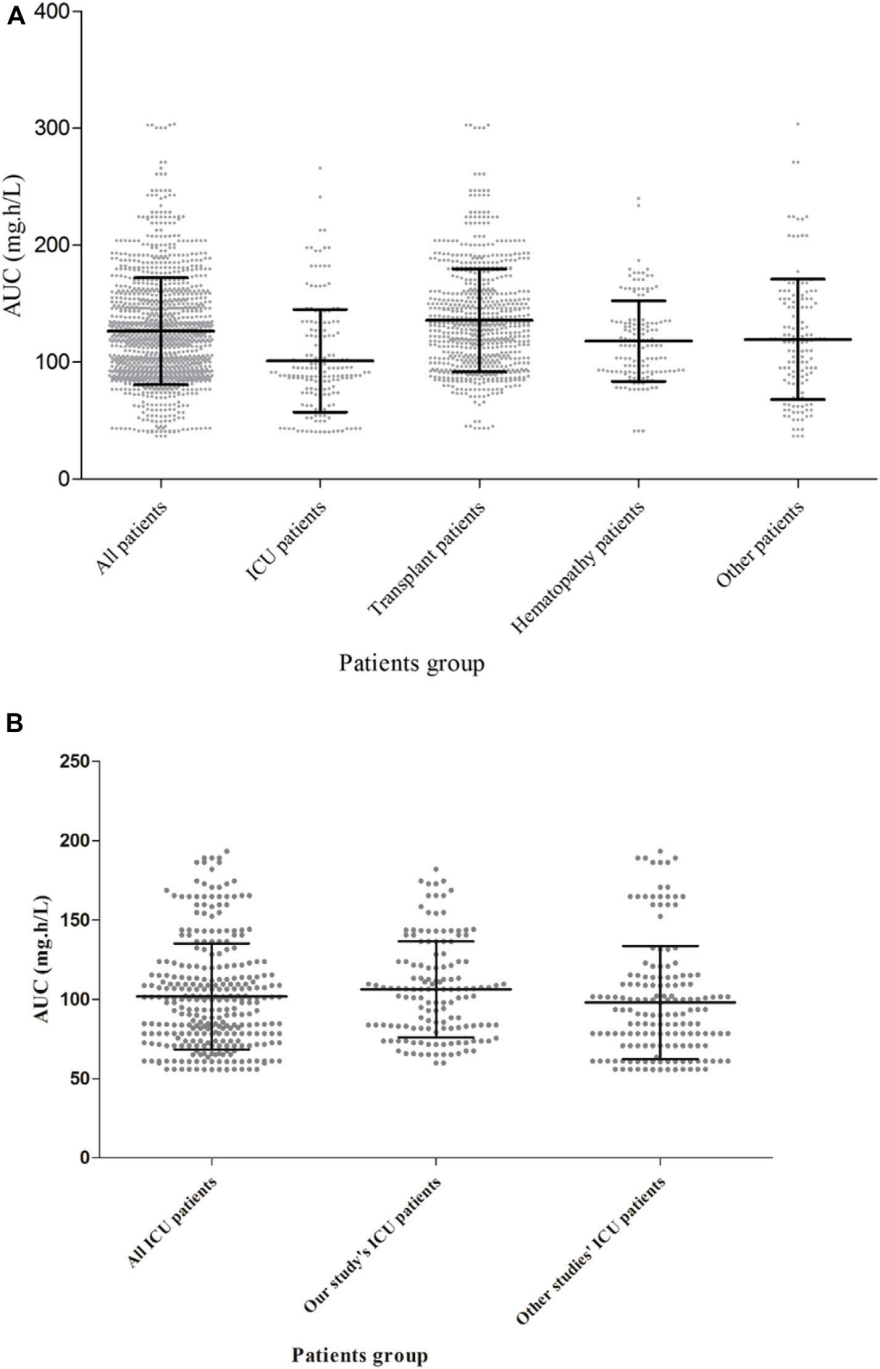
Caspofungin AUC at steady state for different kinds of patients in 299 patients from the teaching hospital **(A)** and intensive care unit-subgroup patients **(B)**. The AUC were different between ICU patients, solid organ transplant patients, hematopathy patients and other patients (*p* < 0.05, Kruskal–Wallis test).

Table 4 also shows the AUC, CL and relative risk factors for caspofungin in ICU patients based on the ICU-subgroup PPK model. We can see the AUC distribution of caspofungin in these ICU patients in Figure 3B. All of them had low ALB concentrations. Detailed information are shown in Supplementary Appendix 3.4.2.

### 3.5 Different caspofungin dosing simulations

#### 3.5.1 Dosing simulations for all patients

**FIGURE 4:**
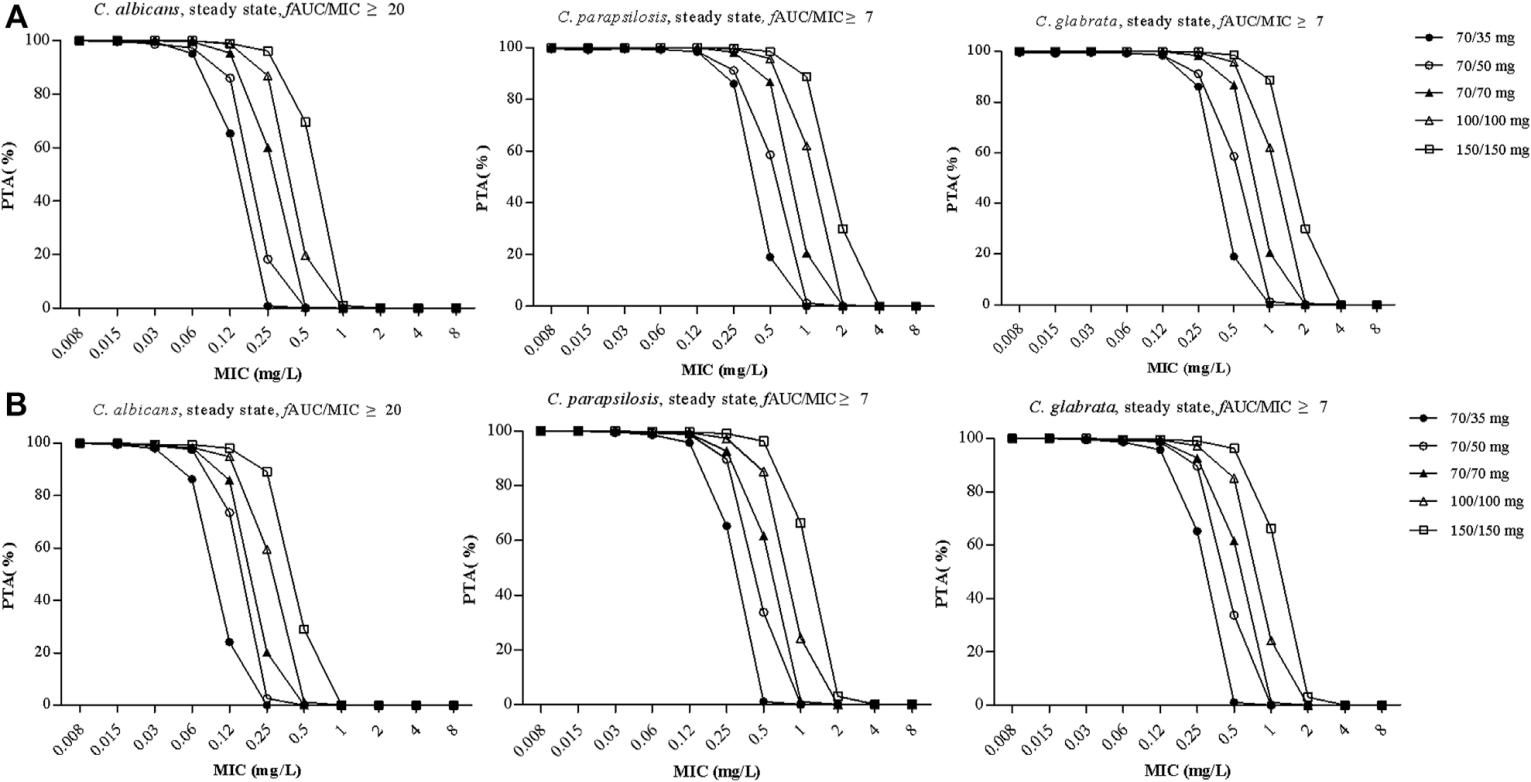
The probability of target attainment (PTA) *versus* MIC for the five simulated caspofungin regimens based on a preclinical target *f*AUC/MIC ratio of ≥20 for *C. albicans*, a target *f*AUC/MIC ratio of ≥7 for *C. parapsilosis* and *C. glabrata* in all patients from the teaching hospital **(A)** and intensive care unit-subgroup patients **(B)**. AUC, area under the concentration time curve; MIC, minimal inhibitory concentration.

Figure 4A shows PTA *versus* MIC for all of the five simulated caspofungin dosage regimens based on a preclinical target *f*AUC/MIC ratio for all patients. For a MIC of 0.12 mg/L for *C. albicans*, the PTAs for caspofungin doses of 70/50 mg and 70/70 mg administered intravenously were 86.1% and 95.5%, respectively. For higher MICs (≥0.25 mg/L), the PTAs for both dosage regimens were <90%. Caspofungin at 150/150 mg achieved a PTA of 96.2% for a MIC of 0.25 mg/L for *C. albicans*. For a MIC of 1 mg/L for *C. parapsilosis*, caspofungin at 150/150 mg achieved a PTA of 88.8%, almost achieved the required PTA. None of the other regimens achieved a PTA of >90%. For a MIC of 0.12 mg/L or 0.25 mg/L for *C. glabrata*, except caspofungin at 70/35 mg did not achieved the required PTA at MIC of 0.25 mg/L, all the other caspofungin dosage regimens achieved the required PTA. Table 5 shows that except caspofungin at 70/35 mg did not achieved the required CFR for *C. parapsilosis*, all the other caspofungin dosage regimens achieved the required CFR both for *C. albicans*, *C. parapsilosis* and *C. glabrata*.

**TABLE 5:** Cumulative fraction of response (CFR) following various caspofungin dosage regimens for all patients^a^ from the teaching hospital and intensive care unit-subgroup patients^b^.

Dosing regimens (mg/day)	C*.albicans*		C.*parapsilosis*		C.*glabrata*	
	All patients	ICU-subgroup patients	All patients	ICU-subgroup patients	All patients	ICU-subgroup patients
70/35	93.5	86.4	88.0	76.4	97.8	96.6
70/50	96.0	94.8	92.8	86.2	98.2	97.8
70/70	98.5	96.4	98.1	93.8	99.0	98.4
100/100	99.2	97.6	99.4	97.4	99.4	98.8
150/150	99.5	98.9	99.8	99.1	99.6	99.2

^a^ All patients include ICU, patients, transplant patients, hematopathy patients and other patients from the teaching hospital.

^b^ The intensive care unit patients include patients from the teaching hospital and the seven literatures.

**FIGURE 5:**
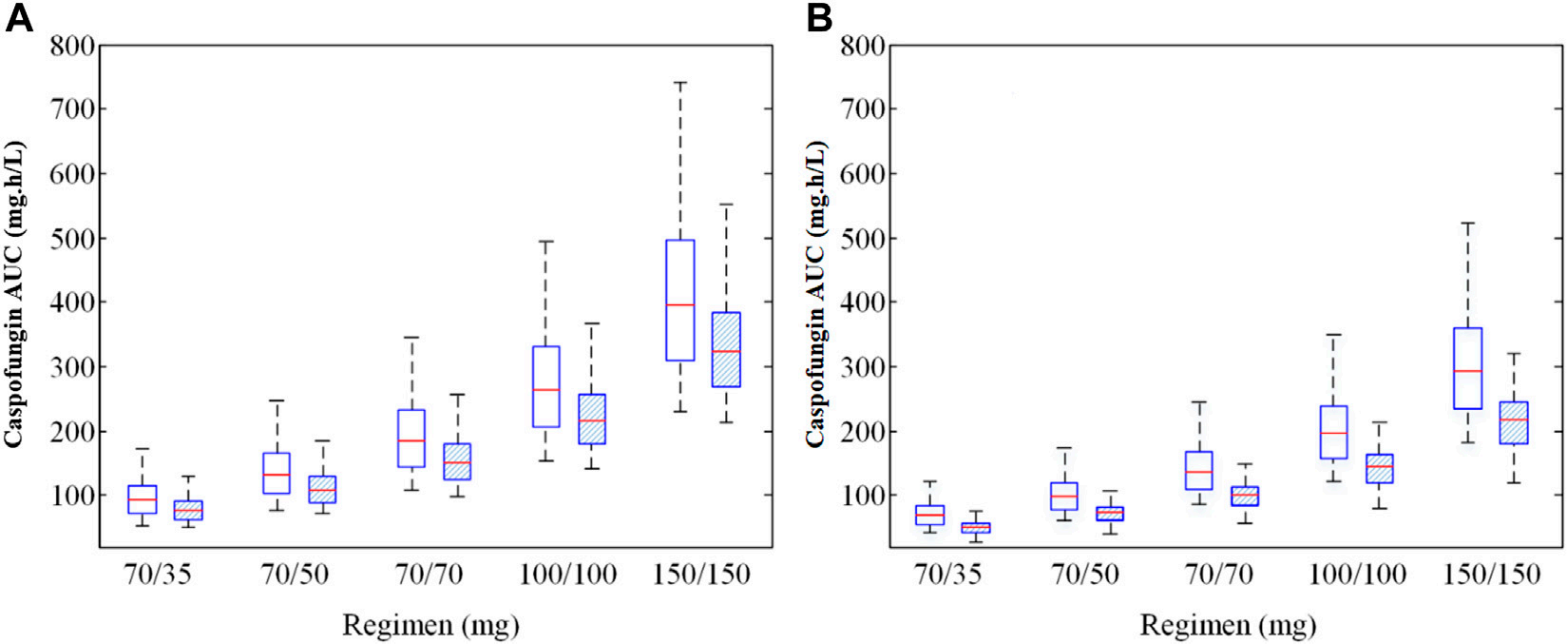
Caspofungin AUC at steady state for all patients from the teaching hospital **(A)** and intensive care unit-subgroup patients **(B)** with different weight groups (WT ≤ 70 kg or >70 kg). 70/35 mg means 70 mg loading dose followed by 35 mg maintenance; 70/50 mg means 70 mg loading dose followed by 50 mg maintenance; 70/70 mg means 70 mg daily; 100/100 mg means 100 mg daily; 150/150 mg means 150 mg daily; AUC, area under the concentration-time curve.

Simulations of alternative dosage regimens based on WT were performed in all patients. AUC_24_ values under different WT conditions (≤70 kg and >70 kg) achieved by different dosage regimens are shown in Figure 5. MCS were applied to the PK/PD targets for the various dosage regimens under different WT conditions. Supplementary Appendix 3.5.1 and Supplementary Table S1 lists the PTA and CFR values stratified by WT for all patients.

#### 3.5.2 Dosing simulations for ICU patients

Figure 4B shows PTA *versus* MIC for ICU patients. For a MIC of 0.12 mg/L for *C. albicans*, the PTAs for caspofungin doses of 70/50 mg and 70/70 mg administered intravenously were 73.5% and 85.9%, respectively. For higher MICs (≥0.25 mg/L), the PTAs for both dosage regimens were <20%. A caspofungin dosage of 100/100 mg achieved a PTA of 94.9% for a MIC of 0.12 mg/L for *C. albicans*. For a MIC of 1 mg/L for *C. parapsilosis*, caspofungin at 150/150 mg could not achieve the required PTA (66.3%). None of the other regimens achieved a PTA of >90%. For a MIC of 0.12 mg/L or 0.25 mg/L for *C. glabrata*, except caspofungin at 70/35 mg did not achieved the required PTA, all the other caspofungin dosage regimens achieved the required PTA. Table 5 shows that except caspofungin at 70/35 mg did not achieved the required CFR for *C. albicans* and *C. parapsilosis*, and caspofungin at 70/50 mg did not achieved the required CFR for *C. parapsilosis*. All the other caspofungin dosage regimens achieved the required CFR both for *C. albicans*, *C. parapsilosis* and *C. glabrata*.

The exposure (AUC_24_) of caspofungin varied markedly between all five dosage regimens and the different WT conditions (*p* < 0.05) for all patients (Figure 5A) and ICU patients (Figure 5B). Supplementary Appendix 3.5.2 and Supplementary Table S2 shows the PTA and CFR stratified by WT in ICU patients.

## 4 Discussion

This is the first study of PPK models for caspofungin in different kinds of patients and especially in ICU patients. By applying established PPK models and the MCS method we were able to successfully identify factors influencing the PK of caspofungin and determine the PTA values for different caspofungin dosage regimens in patients with *Candida* infections or suspected fungal infections and also in patients with different WT values.

The first goal of this study was to determine whether the contemporary dosage regimens for caspofungin can achieve the target PK/PD index against *Candida* isolates in all patients and especially in ICU patients. Caspofungin demonstrate drug exposure-efficacy relationships, and maximum concentration/MIC ratio (C_max_/MIC) and AUC/MIC are proposed PK/PD markers for clinical response (Lewis, 2011). At present, the relationship between caspofungin C_max_ and toxicity remains poorly clarified (Kim et al., 2022). In this study, we only used the *f*AUC/MIC as caspofungin PK/PD target for we could get the *f*AUC_24_/MIC targets both for *C. albicans, C. parapsilosis* and *C. glabrata* (Andes et al., 2010). If C_max_/MIC was used as PK/PD target, we could only get the C_max_/MIC target for *C. albicans* (Lewis, 2011)*.* A previous study performed at another teaching hospital in China found that the mean caspofungin MIC values for *C. albicans*, *C. parapsilosis* and *C. glabrata* were 0.19 mg/L (range 0.032–0.38 mg/L), 1.5 mg/L (0.38–2 mg/L) and 0.25 mg/L (0.047–0.38 mg/L), respectively (Li et al., 2013). For patients with MIC ≥0.25 mg/L with *C. albicans* infections, it is recommended to adjust caspofungin dosage regimen to 70/70 mg (for all patients with WT ≤ 70 kg) or 100/100 mg (for all patients with WT > 70 kg and ICU patients with hypoalbuminaemia). For patients with *C. parapsilosis* infections, the recommended dosage regimen for caspofungin could only achieve the PTA at MIC ≤0.25 mg/L for general patients and at MIC ≤0.12 mg/L for ICU patients. However, the MIC with a range of 0.38–2 mg/L for the hospitalized patients was much higher than 0.25 mg/L or 0.12 mg/L, which suggested that a higher dosage of 150/150 mg was required for patients infected with *C. parapsilosis*. For patients with *C. glabrata* infections, caspofungin recommended dosage regimen (70/50 mg) could achieve the PTA at MIC ≤0.25 mg/L for all patients. However, much higher doses than that should be considered in all patients with WT > 70 kg and in ICU patients with hypoalbuminaemia. For PTA analysis, the relatively low MIC value mentioned above was in line with experimentally determined MIC values in our hospital setting. Similarly, the activity drops off sharply for less susceptible organisms in our hospital setting.

Based on physiological rationality and extensive evidence, WT was incorporated as an important covariate in both PPK models. According to the manufacturer’s suggestion, patients with WT > 80 kg are advised to receive caspofungin at 70/70 mg (Muilwijk et al., 2015). A study of surgical ICU patients showed that lower *C*_min_ concentrations were predicted in patients with WT > 75 kg (Nguyen et al., 2007). Martson *et al.* suggested weight-based dosing of caspofungin in ICU patients for achieving adequate exposure of caspofungin (Martson et al., 2020). They found that the registered caspofungin dose might not be suitable for critically ill patients who were all overweight (120 kg), over 80% of median weight (78 kg), and around 25% of lower weight (50 kg). So a weight-based dose regimen might be appropriate (Martson et al., 2020). These results differ from another study of ICU patients finding that WT had no effect on caspofungin PK (Wurthwein et al., 2012; Muilwijk et al., 2014). Our general PPK model for all patients indicated that an increase in WT would increase both the V and CL of caspofungin, which may lead to decreases in the maximum plasma concentrations and AUC of caspofungin. The same result was found for ICU patients based on the ICU-subgroup PPK model. According to the two PPK models, the caspofungin maintenance dose should be increased for patients with WT > 70 kg.

The general PPK model showed significant associations of patients with hypoalbuminaemia (ALB <35 g/L) with increases in both CL and V (ΔOFV = 77.9), which means the degree of hypoalbumenia had impact on caspofungin pharmacokinetics. A previous study of surgical ICU patients found that the caspofungin *C*_min_ was significantly decreased in patients with ALB <23.6 g/L (*p* = 0.030) (Nguyen et al., 2007). But when the ALB<23.6 g/L was evaluated in the ICU subgroup, it was found that the ALB was not included in the final ICU-subgroup PPK model. Caspofungin was extensively bound to ALB (97%) (Stone et al., 2004). ICU patients often suffer from liver disease, renal insufficiency, profuse bleeding or burns (Pea et al., 2005), which make them much more likely to develop hypoalbuminaemia. However, ALB was not included in the final ICU-subgroup PPK model, since >90% of the ICU patients had ALB <35 g/L, which would have affected the final result. The simulated AUC in the ICU-patients group based on the ICU-subgroup model was lower than that in all patients (*p* < 0.05). The baseline patient characteristics had a little different between all ICU patients, ICU patients from the teaching hospital and ICU patients from other seven studies (Table 1). The WT of other studies’ ICU patients was a little higher than that of our ICU patients. So the simulated AUC of other studies’ ICU patients was a little less than that of our ICU patients. Blood ALB concentrations are known to usually remain high enough to ensure that the unbound fractions of administered drugs remain relatively constant (Nguyen et al., 2007). However, ICU patients often suffer hypoalbuminaemia. Based on the general caspofungin PPK model, ALB was one of the important factors predicted to influence the caspofungin CL. One possible reason is that the protein-binding rate of caspofungin is very high, and hypoalbuminaemia will reduce the binding of caspofungin to ALB, which will increase the CL of caspofungin in patients with hypoalbuminaemia.

Based on the general PPK model, the typical CL for caspofungin in all patients was 0.32 L/h for patients with WT ≤ 70 kg and ALB ≥35 g/L (Table 3). The CL in our study was lower than those found in two studies by Wurthwein *et al.* in general patients: 0.40 L/h (Wurthwein et al., 2013) and 0.46 L/h (Wurthwein et al., 2012). The CL and V values based on the general model were also lower than that reported in ICU patients (0.55 L/h) by Bruggemann *et al.* (Martial et al., 2016). Based on the ICU-subgroup PPK model, the typical CL was 0.56 L/h in ICU patients with WT > 70 kg, which was comparable with the CL (0.55 L/h) in ICU patients found by Martial et al. (Martial et al., 2016). In fact, most of the ICU patients in the other seven studies had WT > 70 kg, so our PPK model for ICU patients was comparable with the PPK model of Martial et al.. Borsuk-De Moor et al. found the typical CL values in ICU patients at days 1, 2, and 3 were 0.563 L/h, 0.737 L/h, and 1.01 L/h, respectively (Borsuk-De Moor et al., 2020). The CL at days 2 and 3 were a little higher than the typical CL of 0.56 L/h by us. Li et al. found in critically-ill Chinese patients, CL of caspofungin was 0.32 L/h (Li et al., 2021), which was lower than the typical CL by us.

Caspofungin clinical treatments might fail due to the low exposure of caspofungin in the patients, and hence the caspofungin exposure needs to be increased. Safety also needs to be considered when increasing the caspofungin exposure, because any harm to the patient should be minimal. Previous studies have shown that in patients with invasive candidiasis, caspofungin was well tolerated at a dosage regimen of 150 mg daily. Betts *et al.* found that with a mean duration of caspofungin therapy of 14.5 days (range, 1–49 days) for the 70/50 mg treatment group and 14.2 days (range, 1–51 days) for the 150 mg treatment group, both caspofungin dosing regimens were effective and well tolerated in patients with invasive candidiasis. No safety concerns were found for caspofungin at a dosage of 150 mg/day (Betts et al., 2009). Cornely *et al.* found that for patients received caspofungin dosage regimen of 150 mg daily for a median of 24.5 days, the treatment was well tolerated without dose-limiting toxicity (Cornely et al., 2011). These studies demonstrating that caspofungin is generally safe at higher doses (Betts et al., 2009; Cornely et al., 2011).

For PPK model establishment, we collected the pooled data of caspofungin plasma samples from patients, and the median number of plasma samples was three for each patient. But the three samples were not from the dense sampling. So one of the limitations of the current study was the lack of dense sampling of caspofungin and the general one-compartment PPK model being largely based on caspofungin plasma samples obtained during the elimination phase. This means that the concentration–time curve was established based mainly on the caspofungin plasma concentrations during that phase. All patients received caspofungin as an intravenous infusion method, and caspofungin can be quickly distributed to the tissues, organs and body fluids of the whole body, and can immediately complete the dynamic balance between transport after intravenous infusion. So the one-compartment model is a good choice for caspofungin. When a two-compartment PPK model was used to fit the data, the OFV was much higher, the residual error was larger, and the GOF was worse than the one-compartment PPK model. Further studies are needed to explore the PK profile of caspofungin in patients with dense sampling. Another limitation of the study was that the ALB and WT variables were kept in a dichotomized fashion, which may take away the power of the ALB and WT distribution. When they were tested as continuous covariates, the OFV value did not decrease and they were not reserved in the PPK model. But when they were transformed into categorical variables, they were included in the analysis and successfully discovered the clinical situations of the real world. On the other hand, the WT distribution of the patients in our study may be a potential study limitation given that there are much heavier patients that receive caspofungin than are described in the present analysis. Further studies should include patients with higher WT to investigate the PK characteristics of caspofungin in them. The PK/PD targets for the various dosage regimens under different WT conditions (WT ≤ 70 kg or >70 kg) were simulated with MCS. Here we divided the WT into two groups according to the WT distribution characteristics of Chinese people. But a more detail weight-based dosing in different grouping situations need to be analyzed in further studies. An analysis of the *f*AUC/MIC associated with clinical success and/or mortality in the patients with confirmed candidiasis would be beneficial given that the current reference for PTA is not from patients. But this is a retrospective study and we cannot get the MIC of these patients. Further prospective studies should focus on this. Only *f*AUC was used in the simulations, but our study found that hypoalbuminemia affects caspofungin exposure, thus using a fixed protein binding value may bias the result, so the absolute AUC analysis should be studied subsequently.

## 5 Conclusion

In conclusion, the PPK model utilized in this study provides a method for predicting caspofungin exposure in all patients, and especially in ICU patients with hypoalbuminaemia for specific dosage regimens. It is recommended that factors such as ALB and WT values should be considered in clinical practice, and monitoring of the caspofungin plasma concentration may be required for ICU patients with hypoalbuminaemia and for patients with significant changes in ALB levels and WT. In order to achieve the PTA, we recommend using a higher caspofungin maintenance dose of 70–150 mg in ICU patients who suffer from hypoalbuminaemia and in patients who are infected with pathogens with higher MICs.

## Data Availability

The original contributions presented in the study are included in the article/Supplementary Material, further inquiries can be directed to the corresponding author.
