# Supplementary material for: The recommended dosage regimen for caspofungin in patients with higher body weight or hypoalbuminaemia will result in low exposure: Five years of data based on a population pharmacokinetic model and Monte-Carlo simulations
Source: Front Pharmacol. 2022 Nov 3;13:993330. doi: 10.3389/fphar.2022.993330 (PMC9669616; doi:10.3389/fphar.2022.993330)
Supplement: Supplementary file 1 [file Presentation1.pdf]

The recommended dosage regimen for caspofungin in patients with higher body weight or hypoalbuminaemia will result in low exposure: 5 years of data based on a population pharmacokinetic model and Monte-Carlo simulations

3.Results

3.1. Patient characteristics, samples and dosing

3.1.1 Data for all patients

The demographic and clinical data of the estimated covariates are shown in Table 1. The mean concentration was 4.32 mg/L (range, 0.1-11.63 mg/L). Seventy-five patients were classified with Child-Pugh score B, but only 31 of the 75 patients actually received the reduced dose (70/35 mg). All of the other 266 patients received the dose regimens of 70/50 mg or 50/50 mg. Eleven patients in the other patients group received the dose regimen of 70/35 mg.

3.1.2 Data for ICU patients

Caspofungin plasma samples for ICU patients were from the teaching hospital and 7 other studies. Thirty-six caspofungin maximum plasma concentrations (C_{max}) were obtained from 27 ICU patients on concomitant continuous renal replacement therapy (CRRT) (Weiler et al., 2013). A PK study of caspofungin in 21 ICU patients obtained the plasma samples on day 3 (+1) of treatment at t = 0 (pre-dose) and 0.5, 1, 2, 4, 6, 8, 12, 16, 20 and 24 h post-infusion, and 18 of them obtained the plasma samples on day 7 (+1) at t = 0 (pre-dose) and 1, 4, 8, 12 and 24 h post-infusion (Muilwijk et al., 2014). A caspofungin population pharmacokinetic (PPK) study in 9 ICU patients undergoing CRRT obtained the full concentration-time profiles, and the plasma samples were taken at 2, 4, 8, 12 and 24 h after the start of caspofungin infusion (Roger et al., 2017). Caspofungin steady-state trough (C_{min}) and C_{max} plasma levels were determined in two studies of caspofungin PK for an ICU patient with liver cirrhosis (Spriet et al., 2011) and a patient during extracorporeal membrane oxygenation (Spriet et al., 2009). Plasma samples from one study of caspofungin low exposure in 20 ICU patients were taken on day 3 (±1 day) just before administration of caspofungin at 1, 2, 3, 4, 6, 8, 12, and 24 h after the start of the infusion (van der Elst et al., 2017). The last one research we brought into the study was a study about caspofungin PK in 6 ICU patients, caspofungin steady-state C_{min} and C_{max} plasma samples were collecter and determined (Sinnollareddy et al., 2015).

3.2. Population pharmacokinetic model

3.2.1 General PPK model

As indicated in Table 2, ALB, mycophenolate mofetil, cyclosporine (CYC), methylprednisolone, WT, CMT, SOP, SOT and ICU were found to have impacts on the caspofungin clearance (CL), while ALB, WT and CMT had impacts on the volume of distribution (V) according to the basic model. Based on 299 patients, we found that ALB, CYC, WT and CMT could be considered as significant covariates for CL, whereas ALB and WT were significant covariates for V in the full model.

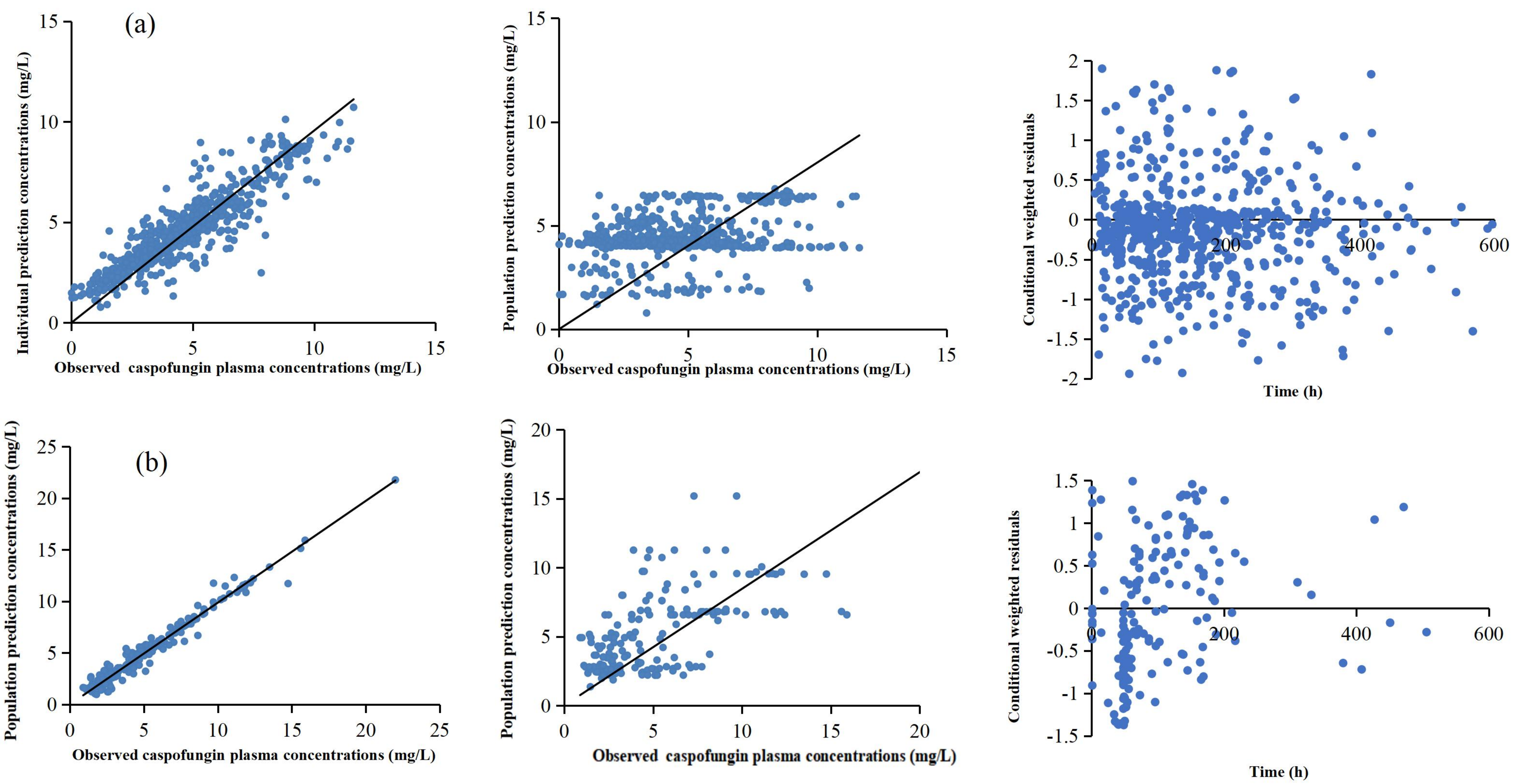

(a)
Individual prediction concentrations (mg/L)
Observed caspofungin plasma concentrations (mg/L)
Population prediction concentrations (mg/L)
Observed caspofungin plasma concentrations (mg/L)
Conditional weighted residuals
Time (h)
(b)
Population prediction concentrations (mg/L)
Observed caspofungin plasma concentrations (mg/L)
Population prediction concentrations (mg/L)
Observed caspofungin plasma concentrations (mg/L)
Conditional weighted residuals
Time (h)

Figure S1 The basic population pharmacokinetic models of caspofungin based on all patients from the teaching hospital (a) and intensive care unit-subgroup patients (b). The diagnostic scatterplots of the caspofungin population pharmacokinetic models from left to right are as follows: individual prediction concentrations versus observed caspofungin plasma concentrations; population prediction concentrations versus observed caspofungin plasma concentrations; and conditional weighted residuals versus time. The diagonal lines in the upper panels represent lines of unit.

3.3. Model validation

The mean values of the overall parameters of the final models were close to that of the bootstrap validation method. The internal verification results showed that the two final models were stable and robust, and the estimated parameter values were reliable. The mean values and 95% confidence intervals of the corresponding parameters are shown in Table 3.

3.4. Pharmacokinetics of caspofungin in all patients and ICU patients

3.4.1 Pharmacokinetics of caspofungin in all patients

Based on the Kruskal-Wallis test, the area under the plasma concentration-time curve (AUC) and CL were different among the four patient groups (Figure 3a, $P < 0.05$). What's more, the relative clinical data (WT, ALB) were different among the four patient groups ($P < 0.05$). As shown in Table 4, 86.3% (44/51) ICU patients had ALB concentrations < 35 g/L and 70.6% (36/51) of them had WT ≤ 70 kg.

3.4.2 Pharmacokinetics of caspofungin in ICU patients

Based on the Kruskal-Wallis test, the AUC and CL were different between these ICU patients and all patients from the general model ($P < 0.05$). The mean AUC in these ICU patients was 101.83 mg·h/L. The AUC of the ICU patients from the teaching hospital was a little higher than that of the ICU patients from other seven studies, and they all had low ALB concentrations.

3.5 Different caspofungin dosing simulations

3.5.1 Dosing simulations for all patients

The PK/PD targets for the various dosage regimens under different WT conditions were simulated with MCS. Table S1 shows the PTA stratified by WT. For *C.albicans*, all the dosage regimens, irrespective of WT, achieved PTA at MIC ≤ 0.06 mg/L. The 70/50 mg of caspofungin did not achieve PTA at MIC = 0.12 and 0.25 mg/L in patients with WT > 70 kg, but achieved PTA > 90% at MIC = 0.12 mg/L in patients with WT ≤ 70 kg. For *C.parapsilosis,* only 150/150 mg of caspofungin achieved PTA at MIC = 1 mg/L with patients' WT ≤ 70 kg. For patients with WT > 70 kg, 150/150 mg of caspofungin did not achieve PTA at MIC = 1 mg/L for patients with *C.parapsilosis* infections. None of the dosage regimens achieved PTA at MIC = 2 mg/L for patients with *C.parapsilosis* infections. For *C.glabrata,* except caspofungin at 70/35 mg did not achieved the required PTA at MIC of 0.25 mg/L, all the other caspofungin dosage regimens achieved the required PTA at MIC = 0.12 mg/L and 0.25 mg/L for all patients with different weight groups (WT ≤ 70 kg or >70 kg).

Except caspofungin at 70/35 mg did not achieved the required CFR for *C.parapsilosis*, all the other caspofungin dosage regimens achieved the required CFR both for *C. albicans, C.parapsilosis and C. glabrata* for all patients with different weight groups.

Table S1 Probability of target attainment (PTA) and cumulative fraction of response (CFR) following various caspofungin dosage regimens for all patients[a] from the teaching hospital with different weight groups (WT ≤ 70 kg or >70 kg).

		PTA (%)									**CFR (%)**
MIC (μg/mL)	*Candida. spp*	0.008	0.015	0.03	0.06	0.12	0.25	0.5	1	2	
**70/35 mg**											
WT ≤ 70 kg	*C.albicans*	100	100	100	95.9	71.2	0.9	0	0	0	**94.6**
	C.parapsilosis	100	100	100	99.4	98.6	89.9	27.4	0	0	**89.7**
	C.glabrata	100	100	100	99.4	98.6	89.9	27.4	0	0	**98.1**
WT > 70 kg	*C.albicans*	100	100	100	97.3	42.8	0	0	0	0	**92.1**
	C.parapsilosis	100	100	100	99.8	99.6	84.1	2.3	0	0	**86.6**
	C.glabrata	100	100	100	99.8	99.6	84.1	2.3	0	0	**98.0**
**70/50 mg**											
WT ≤ 70 kg	*C.albicans*	100	100	100	98.9	89.1	26.1	0	0	0	**97.4**
	C.parapsilosis	100	100	100	99.8	99.6	96.1	67.7	1.9	0	**95.5**
	C.glabrata	100	100	100	99.8	99.6	96.1	67.7	1.9	0	**98.6**
WT > 70 kg	*C.albicans*	100	100	100	99.5	87.8	2.3	0	0	0	**97.2**
	C.parapsilosis	100	100	100	100	99.8	97.8	44.9	0	0	**94.1**
	C.glabrata	100	100	100	100	99.8	97.8	44.9	0	0	**98.5**
**70/70 mg**											
WT ≤ 70 kg	*C.albicans*	100	100	100	99.1	96.7	71.3	0.9	0	0	**98.6**
	C.parapsilosis	100	100	100	99.6	99.7	98.7	90.4	25.7	0	**98.3**
	C.glabrata	100	100	100	99.6	99.7	98.7	90.4	25.7	0	**98.9**
WT > 70 kg	*C.albicans*	100	100	100	99.7	97.9	36.0	0	0	0	**98.5**
	C.parapsilosis	100	100	100	100	100	99.6	84.7	1.4	0	**98.2**
	C.glabrata	100	100	100	100	100	99.6	84.7	1.4	0	**98.9**
**100/100 mg**											
WT ≤ 70 kg	*C.albicans*	100	100	100	99.7	98.3	88.8	26.6	0	0	**99.2**
	C.parapsilosis	100	100	100	100	99.7	99.6	96.7	68.4	1.7	**99.3**
	C.glabrata	100	100	100	100	99.7	99.6	96.7	68.4	1.7	**99.4**
WT > 70 kg	*C.albicans*	100	100	100	100	99.8	85.9	1.9	0	0	**99.3**
	C.parapsilosis	100	100	100	100	100	99.7	97.5	43.1	0	**98.1**

	C.glabrata	100	100	100	100	100	99.7	97.5	43.1	0	**99.1**
**150/150 mg**											
WT ≤ 70 kg	*C.albicans*	100	100	100	99.7	99.6	97.7	75.4	2.6	0	**99.6**
	C.parapsilosis	100	100	100	100	100	99.5	98.1	93.3	37.2	**99.6**
	C.glabrata	100	100	100	100	100	99.5	98.1	93.3	37.2	**99.6**
WT > 70 kg	*C.albicans*	100	100	100	100	100	97.8	50.1	0	0	**99.6**
	C.parapsilosis	100	100	100	100	99.8	98.3	92.3	88.2	5.1	**98.7**
	C.glabrata	100	100	100	100	99.8	98.3	92.3	88.2	5.1	**99.5**

[a] All patients include ICU patients, transplant patients, hematopathy patients and other patients from the teaching hospital.

3.5.2 Dosing simulations for ICU patients

There were big differences in the exposure (AUC_{24}) of caspofungin between all the five dosage regimens and different WT conditions ($P < 0.05$) for all patients (Figure 5a) and ICU patients (Figure 5b). For ICU patients, as show in Table S2, all the dosage regimens, irrespective of WT, achieved PTA at MIC ≤ 0.03 mg/L for *C.albicans.* The 70/50 mg of caspofungin did not achieve PTA at MIC = 0.06 and 0.12 mg/L in patients with WT > 70 kg, but achieved PTA > 90% at MIC = 0.06 mg/L in patients with WT ≤ 70 kg. The 70/70 mg of caspofungin achieved PTA at MIC = 0.12 mg/L for *C.albicans* infections patients with WT ≤ 70 kg but did not achieve PTA at this MIC with patients' WT > 70 kg. For *C.parapsilosis,* none of caspofungin dosage regimens achieved PTA at MIC = 1 mg/L in patients with WT ≤ 70 kg or patients with WT > 70 kg. For *C.glabrata,* except caspofungin at 70/35 mg did not achieved the required PTA at MIC of 0.12 mg/L or 0.25 mg/L, all the other caspofungin dosage regimens achieved the required PTA at MIC = 0.12 mg/L and 0.25 mg/L for all ICU patients with different weight groups (WT ≤ 70 kg or >70 kg).

Caspofungin at 70/35 mg did not achieved the required CFR for *C.albicans* and *C.parapsilosis* for all ICU patients with different weight groups (WT ≤ 70 kg or >70 kg). Caspofungin at 70/50 mg did not achieved the required CFR for *C.albicans* and *C.parapsilosis* for patients with WT >70 kg. Caspofungin at 100/100 mg could achieve the required CFR for *C.parapsilosis* for all ICU patients with different weight groups.

Table S2 Probability of target attainment (PTA) and cumulative fraction of response (CFR) following various caspofungin dosage regimens for intensive care unit-subgroup patients[a] with different weight groups (WT ≤ 70 kg or >70 kg).

		PTA (%)									**CFR (%)**
MIC (μg/mL)	*Candida. spp*	0.008	0.015	0.03	0.06	0.12	0.25	0.5	1	2	
**70/35 mg**											
WT ≤ 70 kg	*C.albicans*	100	100	98.6	92.7	39.4	0	0	0	0	**89.9**
	C.parapsilosis	100	100	100	99.7	98.5	76.2	2.9	0	0	**84.0**
	C.glabrata	100	100	100	99.7	98.5	76.2	2.9	0	0	**97.7**
WT > 70 kg	*C.albicans*	100	100	94.1	74.7	14.2	0	0	0	0	**80.7**
	C.parapsilosis	100	100	98.7	96.8	88.9	42.9	0	0	0	**69.7**
	C.glabrata	100	100	98.7	96.8	88.9	42.9	0	0	0	**94.1**
**70/50 mg**											
WT ≤ 70 kg	*C.albicans*	100	100	99.5	96.2	75.3	1.4	0	0	0	**94.8**
	C.parapsilosis	100	100	100	99.5	98.5	91.9	30.5	0	0	**90.5**
	C.glabrata	100	100	100	99.5	98.5	91.9	30.5	0	0	**98.1**
WT > 70 kg	*C.albicans*	100	100	95.1	86.5	38.1	0	0	0	0	**86.6**
	C.parapsilosis	100	100	98.1	96.7	91.5	70.6	8.2	0	0	**79.0**
	C.glabrata	100	100	98.1	96.7	91.5	70.6	8.2	0	0	**94.8**
**70/70 mg**											
WT ≤ 70 kg	*C.albicans*	100	100	100	98.2	93.1	30.4	0	0	0	**97.6**
	C.parapsilosis	100	100	100	99.6	99.6	96.6	71.5	1.9	0	**96.0**
	C.glabrata	100	100	100	99.6	99.6	96.6	71.5	1.9	0	**98.6**
WT > 70 kg	*C.albicans*	100	100	95.6	93.1	66.7	9.2	0	0	0	**91.5**
	C.parapsilosis	100	100	97.6	98.0	94.8	85.9	38.1	0.2	0	**87.5**
	C.glabrata	100	100	97.6	98.0	94.8	85.9	38.1	0.2	0	**95.8**
**100/100 mg**											
WT ≤ 70 kg	*C.albicans*	100	100	100	99.7	96.9	74.3	1.7	0	0	**98.8**
	C.parapsilosis	100	100	100	100	99.4	98.7	92.3	28.9	0	**98.3**
	C.glabrata	100	100	100	100	99.4	98.7	92.3	28.9	0	**99.0**
WT > 70 kg	*C.albicans*	100	100	97.3	95.3	82.1	36.4	0.1	0	0	**94.6**
	C.parapsilos	100	100	98.2	98.1	96.4	90.4	68.2	0	0	**92.2**

	is										
	C.glabrata	100	100	98.2	98.1	96.4	90.4	68.2	0	0	**96.6**
**150/150 mg**											
WT ≤ 70 kg	*C.albicans*	100	100	100	99.5	98.6	92.9	37.2	0	0	**99.2**
	C.parapsilosis	100	100	100	99.7	99.7	99.5	97.6	76.1	0	**99.4**
	C.glabrata	100	100	100	99.7	99.7	99.5	97.6	76.1	0	**99.4**
WT > 70 kg	*C.albicans*	100	100	97.6	95.1	90.5	66.4	10.3	0	0	**95.8**
	C.parapsilosis	100	100	98.1	97.2	96.5	93.5	82.5	40.5	0	**94.4**
	C.glabrata	100	100	98.1	97.2	96.5	93.5	82.5	40.5	0	**96.8**

[a] The intensive care unit-subgroup patients include patients from the teaching hospital and the seven literatures.

References

Weiler S., Seger C., Pfisterer H., Stienecke E., Stippler F., Welte R., et al. (2013). Pharmacokinetics of caspofungin in critically ill patients on continuous renal replacement therapy. *Antimicrob Agents Chemother* 57(8), 4053-4057. doi:10.1128/AAC.00335-13

Muilwijk E. W., Schouten J. A., van Leeuwen H. J., van Zanten A. R., de Lange D. W., Colbers A., et al. (2014). Pharmacokinetics of caspofungin in ICU patients. *J Antimicrob Chemother* 69(12), 3294-3299. doi:10.1093/jac/dku313

Roger C., Wallis S. C., Muller L., Saissi G., Lipman J., Bruggemann R. J., et al. (2017). Caspofungin Population Pharmacokinetics in Critically Ill Patients Undergoing Continuous Veno-Venous Haemofiltration or Haemodiafiltration. *Clin Pharmacokinet* 56(9), 1057-1068. doi:10.1007/s40262-016-0495-z

Spriet I., Meersseman W., Annaert P., de Hoon J., Willems L. (2011). Pharmacokinetics of caspofungin in a critically ill patient with liver cirrhosis. *Eur J Clin Pharmacol* 67(7), 753-755. doi:10.1007/s00228-011-1066-8

Spriet I., Annaert P., Meersseman P., Hermans G., Meersseman W., Verbesselt R., et al. (2009). Pharmacokinetics of caspofungin and voriconazole in critically ill patients during extracorporeal membrane oxygenation. *J Antimicrob Chemother* 63(4), 767-770. doi:10.1093/jac/dkp026

van der Elst K. C., Veringa A., Zijlstra J. G., Beishuizen A., Klont R., Brummelhuis-Visser P., et al. (2017). Low Caspofungin Exposure in Patients in Intensive Care Units. *Antimicrob Agents Chemother* 61(2). doi:10.1128/AAC.01582-16

Sinnollareddy M. G., Roberts J. A., Lipman J., Akova M., Bassetti M., De Waele J. J., et al. (2015). Pharmacokinetic variability and exposures of fluconazole, anidulafungin, and caspofungin in

intensive care unit patients: Data from multinational Defining Antibiotic Levels in Intensive care unit (DALI) patients Study. *Crit Care* 1933. doi:10.1186/s13054-015-0758-3
